# Cerium-Containing Mesoporous Bioactive Glasses (MBGs)-Derived Scaffolds with Drug Delivery Capability for Potential Tissue Engineering Applications

**DOI:** 10.3390/pharmaceutics14061169

**Published:** 2022-05-30

**Authors:** Irina Atkinson, Ana Maria Seciu-Grama, Simona Petrescu, Daniela Culita, Oana Catalina Mocioiu, Mariana Voicescu, Raul-Augustin Mitran, Daniel Lincu, Ana-Maria Prelipcean, Oana Craciunescu

**Affiliations:** 1 “Ilie Murgulescu” Institute of the Physical Chemistry of the Romanian Academy, 202, Spl. Independentei, 060021 Bucharest, Romania; dculita@icf.ro (D.C.); omocioiu@icf.ro (O.C.M.); voicescu@icf.ro (M.V.); raul.mitran@gmail.com (R.-A.M.); daniel.lincu1113a@gmail.com (D.L.); 2 National Institute of Research and Development for Biological Sciences, 296, Spl. Independentei, 060031 Bucharest, Romania; annastanciuc@gmail.com (A.-M.P.); oana_craciunescu2009@yahoo.com (O.C.)

**Keywords:** bioactive glass, tissue engineering, drug delivery, ceria

## Abstract

Finding innovative solutions to improve the lives of people affected by trauma, bone disease, or aging continues to be a challenge worldwide. Tissue engineering is the most rapidly growing area in the domain of biomaterials. Cerium-containing MBG-derived biomaterials scaffolds were synthesized using polymethyl methacrylate (PMMA) as a sacrificial template. The obtained scaffolds were characterized by X-ray powder diffraction (XRPD), infrared spectroscopy (FTIR), and scanning electron microscopy (SEM). The Ce^4+^/Ce^3+^ ratio in the scaffolds was estimated. In vitro testing revealed good cytocompatibility of the investigated scaffolds in mouse fibroblast cell line (NCTC clone L929). The results obtained regarding bioactivity, antibacterial activity, and controlled drug delivery functions recommend these scaffolds as potential candidates for bone tissue engineering applications.

## 1. Introduction

There are over 1 million cases of bone defects and diseases reported annually. These are due to trauma, congenital anomalies, osteoporosis, bone bacterial infections (e.g., osteomyelitis), and tissue resection that require surgical procedures employing artificial or autologous bone grafting techniques as a treatment [1].

The design and engineering of synthetic scaffolds with ordered architectures are major areas of biomaterial research. They are also important subjects for regenerative medicine as they are able to overcome the limitations associated with current treatments, e.g., immunological rejection and transmitting infectious diseases. Scaffolds have been used for various tissue engineering applications such as bone, cartilages and ligaments, vascular tissues, and as carriers for the controlled delivery of bioactive agents [2]. They mainly consist of bioactive ceramics and glasses, natural or synthetic polymers, and composites of these [3,4,5].

An ideal scaffold for bone regeneration applications must have sufficient porosity to support bone growth, biocompatibility, cytocompatibility, adequate mechanical properties, biodegradability, and bioactivity, a key property to promote osseointegration through the formation of a layer of hydroxyapatite (HA) [6,7]. In addition to these requirements, the delivery of therapeutic agents into the damaged bone tissues has a great importance. Local antibiotic delivery has many advantages over usual administration routes in bone infection therapy: high drug concentration at the diseased site, prolonged and controlled drug delivery, and the reduced adverse side effects of the systemic doses [8].

Mesoporous bioactive glasses (MBGs) are used in synthetic scaffold preparation due to their outstanding properties, e.g., optimal surface area, pore-volume, and ability to induce in vitro HA mineralization [9,10,11]. MBGs can impart multiple therapeutic actions supporting bone tissue regeneration. They concomitantly provide a localized treatment of the diseased bone through the controlled release of drugs or inorganic therapeutic agents. In addition, the dissolution products such as Si^4+^ and Ca^2+^ from the MBGs can improve cell differentiation and proliferation, facilitating new bone formation and having a positive effect on the expression of genes [12]. Introducing specific metallic ions into the MBG structure (e.g., Zn^2+^, Cu^2+^, Ga^2+^, Ce^3+^, and Ce^4+^) can improve their properties that induce antibacterial action or act as tumor therapy [13]. In this regard, it has been demonstrated that cerium-containing MBGs exhibited great potential considering their antioxidant, antibacterial, anti-inflammatory, and pro-osteogenic activities for a variety of biomedical devices [14]. Moreover, cerium can mimic enzymes such as superoxide dismutase, catalase, and oxidase, and change its oxidation states (Ce^4+^ and Ce^3+^) in physiological fluids, reducing reactive oxygen species (ROS) essential to retain healthy biological functions [15]. The antibacterial effect of cerium oxide is still controversial. Some reports showed no antibacterial activity [16] and others suggested that cerium oxide can impart an antibacterial effect through the oxidative stress of components of the cell membrane of the bacteria [17]. Some studies reported that cerium-containing bioactive glasses exhibit antibacterial effects towards *Escherichia coli* [18] and *Staphylococcus aureus* [19].

Due to their mesoporosity, MBGs possess lower mechanical properties that limit their applications. The fabrication of MBG-based scaffolds is a challenge; macroporosity must be present in the manufactured product to facilitate cell attachment and tissue in-growth. Polymers have been widely used as sacrificial templates for the fabrication of tissue engineering scaffolds [20,21]. The sacrificial template is extracted from the partially consolidated matrix to generate macropores. Among several choices of polymers, poly methyl methacrylate (PMMA) has been frequently explored as an implant material in biomedical applications. PMMA acrylic bone cement has been used extensively in surgical fixation of artificial joints for more than 50 years. It was first used in orthopedics and is credited to Dr. John Charnley who used “dental acrylic” in 1958 for a total hip arthroplasty [22]. Han et al. [23] obtained macro-mesoporous Ti-BGs/PMMA scaffolds with enhanced mechanical strength, good antimicrobial activity, and biocompatibility.

This study aimed to obtain MBG-derived scaffolds containing cerium up to 3 mole %, using PMMA as sacrificial template with antibacterial activity and drug delivery capability. Drug-loaded bone scaffolds offer the facility of both treatment and also the replacement of the damaged bone tissues. Recently, scaffolds used as drug delivery systems have attracted considerable interest and represent the next generation of tissue engineering. The effect of cerium on the bioactivity, cytocompatibility, antibacterial activity, and drug release was evaluated.

## 2. Materials and Methods

### 2.1. Preparation of Cerium-Containing MBGsScaffolds

MBG-based biomaterial scaffolds were prepared using PMMA as sacrificial template. MBG precursor sols were directly used to obtain the investigated scaffolds. In brief, Cerium-doped MBGs in the 70SiO_2_-(26-x) CaO-4P_2_O_5_-xCeO_2_ system (where x stands for 0, 1, and 3 moles %) were synthesized as described in paper [24]. Pluronic P123 (Sigma Aldrich), was used as structure directing agent.

For MBG polymer scaffolds synthesis, PMMA (Alfa Aesar) with a molecular weight of 550,000 and a density of 1.18 g/cm^3^ was used as a pore former for sacrificial template technique. PMMA (15%) was dissolved in ethanol and water mixture. Equal volumes of MBG solution and the polymer mixture were mixed to obtain the scaffold materials. The obtained mixtures were transferred into removable molds and kept for 7 days at room temperature. Afterwards, the as-prepared scaffolds were removed from the molds and thermally treated at 600 °C, according to DTA results [25], with a heating rate of 1°/min and 2h plateau at maximum temperature to remove the PMMA template, Pluronic P123, and the nitrates. Scaffolds with a diameter of 10 mm and a height of 5 mm were obtained. They were labeled as follows: S0Ce, S1Ce, and S3Ce respectively.

### 2.2. Characterization of Cerium-Containing MBGs Scaffolds

The absorption measurements were recorded with a Perkin Elmer Lambda 35 Spectrometer with integrating sphere in 900–200 nm range using: data interval, 1 nm; scan speed, 60 nm/min; slit, 4 nm; sample holder at 8° wedge; and a certified reflectance standard. To estimate the cerium concentration in scaffolds, a least-squares iterative curve fitting was performed with Gaussian bands using the Peak Fit Analysis Program (Sea-Solve, MA, USA). The areas of all bands assigned to a given concentration were summed up and divided by the total area in order to obtain the contribution of cerium(Ce^4+^/Ce^3+^) as adapted from Ref. [26].

Nitrogen adsorption–desorption isotherms at 77 K were recorded on a Micromeritics ASAP 2020 analyzer (Norcross, GA, USA). The samples were degassed at 300 °C for 4 h under vacuum before analysis. Specific surface areas (SBET) were calculated according to the Brunauer–Emmett–Teller (BET) equation, using adsorption data in the relative pressure range between 0.05 and 0.30. The total pore volume was estimated from the amount adsorbed at the relative pressure of 0.99. The average pore diameters were calculated from the adsorption data using the BJH (Barrett–Joyner–Halenda) model.

Powdered X-ray patterns (XRPD)of the scaffolds were recorded using a Rigaku Ultima IV diffractometer (Rigaku Co., Tokyo, Japan) in parallel beam geometry equipped with CuKα radiation (wavelength 1.5406 Å) in 2θ range between 10° to 70° with a speed of 2°/min and a step size of 0.02°. PDXL software (Version 1.8) from Rigaku, connected to ICDD database was used for phase identification.

Fourier transform infrared (FTIR) spectroscopy was performed with a Nicolet Spectrometer 6700 FTIR, within 400–4000 cm^−1^ range, in transmittance mode.

FEI Quanta 3D FEG microscope (FEI, Brno, Czech Republic) equipped with an Octane Elect EDS system was used to study the morphology of the investigated samples that were placed on a double-sided conductive carbon tape and scanned without coating.

In vitro bioactivity of the obtained scaffolds was evaluated by immersing the samples in the simulated body fluid (SBF) as proposed by Kokubo et al. [27] at 37 °C and pH 7.33 for a different time interval (up to 21 days). The scaffold/SBF ratio was 1 g to100 mL SBF [24]. Once removed from the incubation solution, the samples were washed with deionized water and dried at 70 °C for 24 h. The formation of HA on the surface of scaffolds was further examined by XRPD, FTIR, and SEM analysis.

Cell culture testing was performed in a mouse fibroblasts cell line (NCTC clone L929) (ECACC) using the extract method. Cells were cultured in Minimum Essential Medium (MEM) supplemented with 10% (*v*/*v*) fetal bovine serum (FBS), 2 mM L-glutamine, and 1% (*v*/*v*) antibiotic mixture of penicillin–streptomycin–neomycin (PSN) in humid atmosphere with 5% CO_2_, at 37 °C, until sub confluence. The scaffolds were incubated in culture medium at 37 °C, for 24 h and then, filtered using 0.22 µm Millipore membranes (Millipore, Merck, Germany) to obtain sterile stock solutions. For the experiment, L929 cells were seeded at a density of 4 × 104 cells/mL, in 96-well microplates, cultivated in MEM supplemented with FBS and PSN and incubated in humid atmosphere with 5% CO_2_, at 37 °C for 24 h, in order to allow cell adhesion to the plastic substrate. Then, the culture medium was replaced with fresh medium containing serial dilutions of sterile stock solutions and the incubation continued in standard conditions, for 48 and 96 h. The cells incubated in culture medium without sample served as control. At the end of each incubation period, both cells and the harvested conditioned medium were used to assess the cytocompatibility of the obtained scaffolds by two complementary assays.

First, the cell viability was assessed by MTT assay based on tetrazolium salt reaction with mitochondrial dehydrogenases in viable cells, as previously described [28]. Briefly, the cells were rinsed with phosphate buffered saline solution (PBS), pH 7.4 and incubated in 0.25 mg/mL 3-(4,5-dimethyl-thiazol-2-yl)-2,5-diphenyltetrazolium bromide (MTT) solution in culture medium at 37 °C, for 3 h. Then, the culture medium was harvested, and isopropyl alcohol was added to each well, to solubilize the formazan crystals. After incubation at room temperature, for 15 min, under gently stirring, the absorbance was read at 570 nm using a Sunrise microplate reader (Tecan, Austria). The values were directly correlated to the number of metabolically active cells. Cell viability was expressed as a percentage of the value of control cells considered 100% viable. The tests were performed in triplicate.

In the harvested conditioned medium, the lactate dehydrogenase (LDH) activity was assessed using CytoTox 96 Non-Radioactive Cytotoxicity Assay kit (Promega, UK). The assay determined the activity of cytoplasmic LDH released into the culture medium by membrane damaged cells and the used protocol was previously described [29]. Briefly, 50 μL of conditioned medium was incubated with 50 μL of mixed reaction solution in the dark, at room temperature, for 30 min. Then, 50 μL of stop solution was added and the absorbance was read at 490 nm using a Sunrise microplate reader (Tecan, Austria). The values were proportional to the number of cells that have lost their cell membrane integrity and, therefore, their viability. The tests were performed in triplicate.

The antibacterial activity of the prepared scaffolds was evaluated against *S. aureus* (ATCC 25923). Standardized microbial suspensions with a density of 0.5 McFarland were prepared from 24-hour cultures of *S. aureus* grown on nutrient agar (TSA) under aerobic conditions at 37 °C. The bacterial inoculum was adjusted to a final concentration of 1.5 × 108 colony forming units per mL (CFU/mL) in each well, and the plate was incubated at 37 °C. After 24 h, the absorbance of the supernatant was measured at 600 nm to determine the bacterial viability using a Sunrise plate reader (Tecan). The following equation was used to calculate the relative microbial viability:

Relative bacterial viability = OD sample/OD reference × 100(1)

The bacterial viability was also evaluated after cultivation in the presence of samples loaded with VAN. After 24 and 48 h, the samples were washed with PBS in order to remove the bacterial cells that were not attached, and prior to further processing, sonicated for 10 min. The obtained suspensions were serially diluted and further seeded on nutrient agar (BHI). After 24 h, the seeded Petri dishes were photographed with a 12-Megapixel camera (iPhone 7, Apple Inc., Los Altos, CA, USA). Further, these images were analyzed for the number of microbial colonies (ImageJ, NHS, LOCI, University of Wisconsin USA).

In vitro release capability of the obtained scaffolds was investigated using Vancomycin hydrochloride (VAN) (Sigma Aldrich, St. Louis, MO, USA) loaded onto the inorganic carriers through the incipient wetness impregnation method [30]. The scaffolds were added to a 100 gL^−1^ aqueous solution of VAN. The resulting slurry was mechanically stirred for 10 min and then the solvent was removed under vacuum for 2 h. The resulting drug-loaded scaffolds are denoted “VAN@Carrier”, where “Carrier” denotes the inorganic matrix.

The drug release experiments were performed in 0.1 M phosphate buffer solution (PBS, Boston, MA, USA), at pH 7.4 and 37 °C. In sink conditions were maintained, by using a total VAN concentration of 0.15 gL^−1^ in each experiment. The corresponding quantity of either pure drug or drug-loaded materials was added to a cellulose dialysis bag (molecular weight cutoff 10,000 Da, Sigma Aldrich, St. Louis, MO, USA) together with 0.5 mL PBS. The dialysis bag was sealed and immediately introduced into the release medium. Insitu UV-Vis analysis was carried out for 24 h and the results were used to calculate the VAN concentration versus time release profiles.

Thermogravimetric analyses (TGA) coupled with differential thermal analyses were carried out using a Mettler Toledo TGA/SDTA851e thermogravimeter, under 80 mL min^−1^ synthetic air flow, at a heating rate of 10 °C min^−1^. In situ UV-Vis analyses were performed using an Agilent Cary 60 spectrometer equipped with a fiber optic probe for in situ measurements.

Experimental data were reported as mean ± standard deviation (SD) (*n* = 3). Statistical analysis of the data was performed using Student’s *t*-test on each pair of interest. Differences were considered statistically significant at *p* < 0.05.

## 3. Results and Discussion

### 3.1. UV-Vis

**Figure 1:**
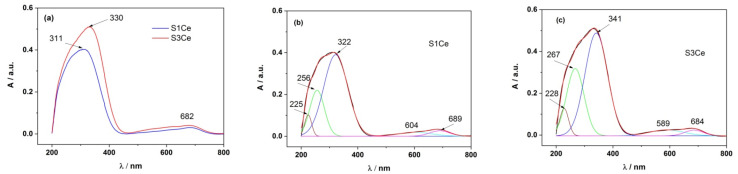
Absorption spectra S1Ce and S3Ce sample (**a**); Deconvolution into Gaussian functions of absorption spectra of S1Ce sample (**b**), and S3Ce sample (**c**).

Absorption spectra of S1Ce and S3Ce scaffolds thermally treated at 600 °C measured in the range of 200–800 nm are shown in Figure 1a. In the 200–400 nm absorption range, specific to the Ce^4+^ and Ce^3+^ absorption, one major absorption peak is observed: a broad band at ~311 nm for S1Ce, and at ~330 nm for S3Ce [31]. As can be observed, the absorption bands of Ce^4+^ and Ce^3+^ are overlapped and the contribution of each of them requires them to be clearly separated in the absorption spectrum. In addition, the contribution of PMMA in the scaffolds and especially the PMMA particles dispersion within the scaffolds, which may lead to changes in the scaffolds morphology as the cerium composition increases, is considered [25]. In this context, for the structure of the absorption band of Ce^4+^ and Ce^3+^, the absorption spectrum of each sample was deconvoluted into Gaussian functions in order to clearly separate the contribution of Ce^4+^ from the one of Ce^3+^, Figure 1b,c. The positions of the maximum of the absorption band of Ce^4+^ and Ce^3+^ as well as their percentage in the scaffolds, are presented in Table 1. There is a clear difference in the percentage of Ce^4+^ in the scaffolds when these are doped with cerium and thermally treated at 600 °C (34.85% Ce^4+^ for S3Ce and 19.79% Ce^4+^ for S1Ce) therefore, changes in the cerium oxidation state occur. The weak and broad absorption bands between 500–750 nm may be attributed to small impurities into the scaffolds that arise from thermal treatment or may be related to the existence of n→π* transitions of PMMA, that occur at high wavelengths.

### 3.2. Textural Characterization

**Figure 2:**
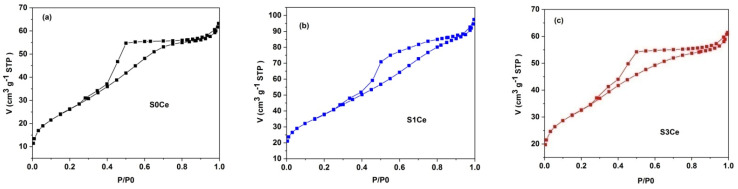
Nitrogen adsorption/desorption isotherms of the investigated samples: (**a**) S0Ce; (**b**) S1Ce, and (**c**) S3Ce.

The nitrogen adsorption–desorption isotherms of the S0Ce–S3Ce scaffolds thermally treated at 600 °C for 2 h showed type IV isotherm patterns according to IUPAC classification (Figure 2) [32]. The occurrence of type H2 hysteresis loops indicates the presence of significant pore network effects. This is characteristic for disordered porous materials, which consist of cavities connected to each other by constrictions [33]. The pore diameters range between 2 and 10 nm with an average value of ~4 nm for S0Ce and S3Ce, whereas for S1Ce the pore size distribution is slightly wider, between 2 and 15 nm, with an average value of ~4.6 nm. The values of the BET surface area are between 97and 137m^2^/g lower than those of the MBGs reported in the literature [34] and in our previous study [23]. Varini et al. [35] reported BET surface areas between 94 and 150 m^2^/g of the alginate-Ce-MBGs beads, without compromising the bioactivity and cells proliferation. Table 2 summarizes the specific surface area, average pore diameter, and total pore volume.

### 3.3. In Vitro Bioactivity Assessment

The formation of the HA layer on the surfaces of the scaffolds in contact with SBF solution is an important feature in order to estimate the ability of the material to form a natural bonding to the surrounding tissues. All prepared samples were immersed in SBF for different periods of time up to 21 days. The formation of the HA layer on the scaffold surfaces was evaluated by SEM, XRPD, and FTIR analyses.

**Figure 3:**
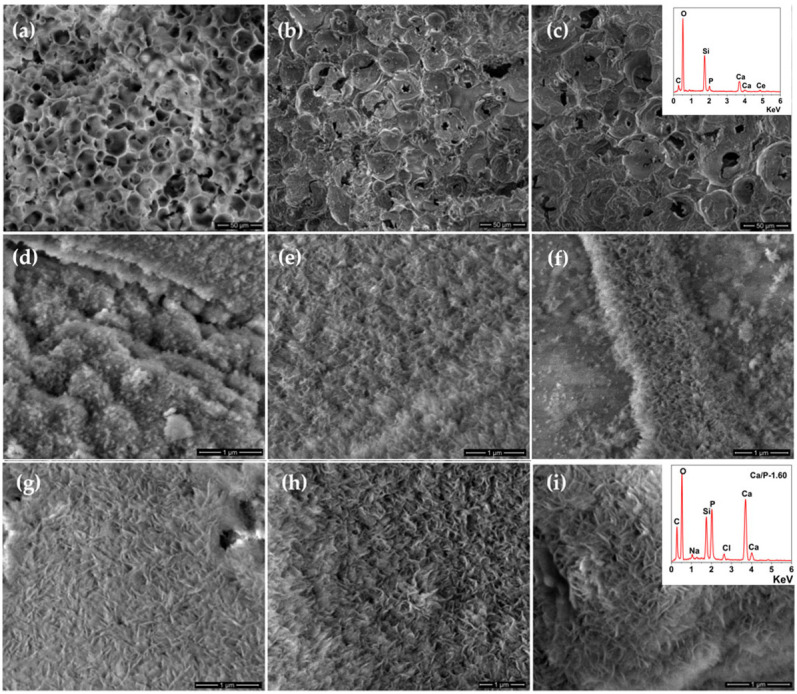
SEM images of S0Ce-S3Ce set before immersion in SBF; (**a**) S0Ce; (**b**) S1Ce; (**c**) S3Ce (EDX inset graph); after 7 days immersion in SBF; (**d**) S0Ce; (**e**) S1Ce and (**f**) S3Ce, and after 21 days immersion in SBF; (**g**) S0Ce; (**h**) S1Ce, and (**i**) S3Ce (EDX inset graph).

The morphology of the obtained scaffolds was analyzed before and after immersion in SBF for 21 days by SEM (Figure 3). Figure 3a–c show the pore morphology, size, and distribution of the scaffolds before immersion in SBF. As can be seen, macropores appropriate for cell accommodation, proliferation, and vascularization were confirmed to be introduced into all of the samples using the PMMA template. Besides macroporosity, mesoporosity plays a significant role in bone regeneration, especially in the release of the biological agents and enhancing the surface activity and bioactivity [36].

The SEM images after immersion in SBF for 7 and 21 days, respectively, revealed the formation of the HA layer on the scaffold’s surfaces. All surfaces exhibited a typical apatite-like semi-crystalline phase in the form of flake-like crystals as was confirmed by XRPD. The formation of the HA layer on the surface of scaffolds was also confirmed by EDX analysis, Figure 3i (inset graph). The Ca/P of the HA precipitate in the case of S3Ce was 1.60 closed to the standard Ca/P ratio of 1.67 of stoichiometric HA.

Additionally, the formation of the HA layer on the scaffold surfaces was also monitored by X-ray powder diffraction and Fourier transforms infrared spectroscopic techniques.

**Figure 4:**
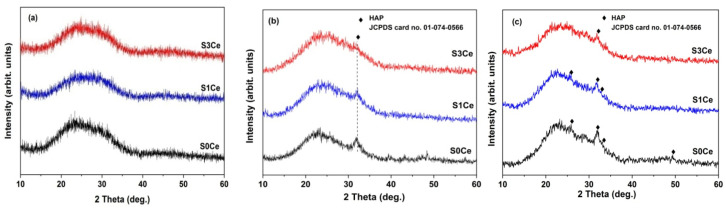
XRD patterns of the S0Ce–S3Ce samples before (**a**) 7 days immersed in SBF; (**b**) and 21 days immersed in SBF (**c**).

Figure 4 illustrates the XRD patterns of the scaffolds before and after immersion in SBF for 7 days and 21 days, respectively. The XRPD patterns of the S0Ce–S3Ce samples thermally treated at 600 °C exhibit a diffraction halo between 15° and 40° (2θ) with the center at 26°, confirming their amorphous structure. XRPD patterns of the samples after immersion in SBF for 7 days (Figure 4b) reveal the presence of low crystalline HA. After 21 days of immersion (Figure 4c), the characteristic diffraction lines of HA at 25.80° (002) and 31.79° (211), 32.78° (300), and 49.93° (213) in agreement with data from JCPDS card no. 009–0432 were observed for the scaffold without cerium. The bioactive performance of the scaffolds decreased when increasing the cerium content.

**Figure 5:**
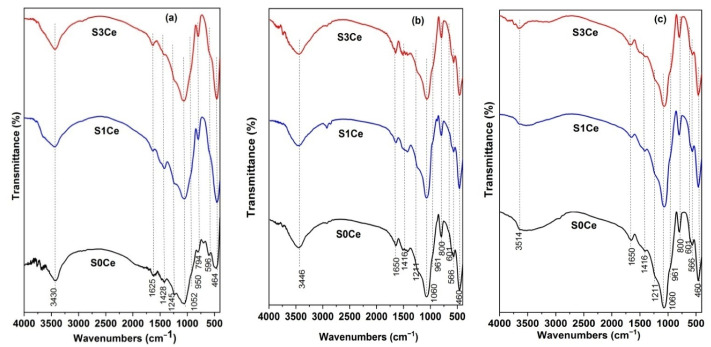
FTIR spectra of the samples before immersion in SBF. (**a**) 7 days immersed in SBF; (**b**) 21 days immersed in SBF (**c**).

Figure 5 shows the FTIR spectra of the S0Ce-S3Ce samples before and after immersion in SBF for 7 and 21 days, respectively. For all samples before immersion in SBF (Figure 5a), the band at 465 cm^−1^ assigned to Si–O–Si bending vibration modes was observed [37]. The Si–O–Si asymmetric stretching vibration mode was observed at 1050 cm^−1^, while the band at 790 cm^−1^ is associated with Si–O–Si symmetric stretching. In the case of S0Ce sample the band located to 590 cm^−1^ corresponds to a phosphate group. The band noticed at about 1245 cm^−1^ was assigned to the Si–O–Si bending mode. The bands observed at 3430 and 1625 cm^−1^, respectively, are due to the stretching and bending modes of absorbed water.

Figure 5b,c depict the FTIR spectra of the scaffolds after immersion in SBF for 7 and 21 days, respectively. After 7 and 21 days of immersion the new bands were observed at 565, 601, and 960 cm^−1^, respectively, characteristic of phosphate groups (PO_3_^−4^), and at around 800 cm^−1^ that corresponds to the Si–O–Si bending mode of orthosilicate SiO_4_^4−^ [38,39]. In the case of the scaffolds immersed for 21 days in SBF (Figure 5c), the vibration band located at 3514 cm^−1^ is assigned to the stretching vibration of the OH group of HA [40].

A detailed study regarding the influence of cerium on the bioactivity was published by Lusvardi et al., [41], where it was concluded that cerium addition retarding the HA layer formation, mainly due to the formation of insoluble crystalline CePO_4_, competitive with HA and the increase in chemical durability. Furthermore, this effect is correlated with the ceria content in the sample: up to 1 mole% HA formation was noticeable after 7−14 days, while the higher ceria content up to 5.3 mole %, the formation of HA was delayed up to 28 days [42].

### 3.4. In Vitro Cytocompatibility Testing

Cell culture assays are the main in vitro tools to predict the biological response of the host organism to a biomaterial that can be performed by direct tests, in the presence of the synthesized material or indirect ones (MTT and LDH) in which filtered extracts of materials are added to the cell culture [43].

**Figure 6:**
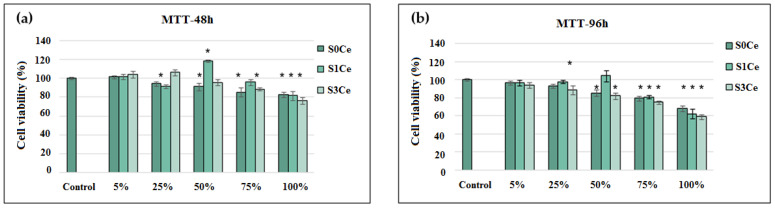
Viability of NCTC L929 cells cultivated in the presence of different concentrations of scaffold extracts evaluated by MTT assay after 48 h (**a**) and 96 h (**b**) of incubation. The results were calculated as mean of three determinations ± SD and reported to the control culture (cells cultivated without sample) considered 100% viable; * *p* < 0.05 compared to control (untreated cells).

In vitro cytocompatibility of the prepared scaffolds was first investigated by MTT assays. The obtained results are presented in Figure 6. After 48 h of incubation, in the presence of different L929 fibroblasts, showed high viability values (78–119%) for all tested concentrations of S0Ce–S3Ce extracts. The cell viability was similar to that of the control (100%) in the presence of 5% S0Ce and S3Ce and then, decreased in a dose-dependent manner, but the values were above 80%, indicating scaffolds’ cytocompatibility in the range of tested concentrations. The concentration of 50% S1Ce extract corresponded to a value of 119% cell viability, revealing a significant (*p* < 0.05) stimulation of cell proliferation compared to control cells, while higher concentrations of S1Ce extract decreased the cell viability values down to 81%. Similar results were observed after 96 h of incubation in the presence of the investigated scaffold extracts. However, the cell viability varied between 80–106% for extract concentrations of 5–75%, excepting the 75% S3Ce sample. At a 100% extract concentration, the moderate cytocompatibility of tested extracts was observed with cell viability values of 75.17% (S0Ce), 72.39% (S1Ce), and 66.51% (S3Ce). The cell viability percentages higher than 70% is the required minimum value for considering biomaterials as non-cytotoxic, according to the international standard ISO 10993-5: 2009-Biological evaluation of medical devices: The tests for in vitro cytotoxicity [44]. Naganuma et al. [45] established that cell proliferation of cerium-doped materials is correlated with the cerium oxidation state (Ce^3+^ vs. Ce^4+^), and that Ce^3+^ ions inhibit cell proliferation and Ce^4+^ ions promote cell proliferation.

**Figure 7:**
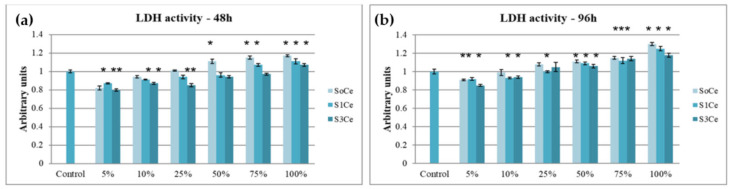
LDH activity of L929 cells cultivated in the presence of different concentrations of scaffold extracts after 48 h (**a**) and 96 h (**b**) of incubation. The results were calculated as mean of three determinations ± SD and reported to the control culture (cells cultivated without sample) considered 1 in arbitrary units (a.u.); * *p* < 0.05 compared to control (untreated cells).

The cytocompatibility of different concentrations of scaffold extracts was also tested by LDH assays. The obtained results are presented in Figure 7. After 48 h of incubation, the activity of LDH released in the culture medium varied in the range of 0.8 a.u. for 5% S3Ce and 1.17 a.u. for 100% S0Ce. All tested samples and concentrations presented values near to that of the control cells (1 a.u.). The highest LDH values were observed for S0Ce at concentrations between 10–100%. At 96 h of cultivation, slightly higher values were recorded, increasing from 0.86 a.u. for 5% S3Ce up to 1.3 a.u. for 100% S0Ce, in a dose-dependent manner. These results confirmed that the prepared scaffolds were cytocompatible, in accordance with the MTT assay.

### 3.5. Antibacterial Activity

Developing materials for regenerative medicine applications to overcome bacterial contamination is a critical and emerging area of biomaterials research. *S. aureus* is the most common pathogen causing bone and joint infections such as infections of prostheses, osteomyelitis, and septic bursitis [46]. Implant-associated infection is a notable clinical problem and is characterized by bacterial adhesion, colonization, and biofilm development. The prosthetic infections place a severe burden on the health service through prolonged hospitalization for antibiotic therapy, prosthesis removal, and replacement. *S. aureus* accounts for 80–90% of cases of pyogenic osteomyelitis, an inflammation of the bone, which can be due to biofilm formation, causing increased bone resorption and reactive bone formation.

**Figure 8:**
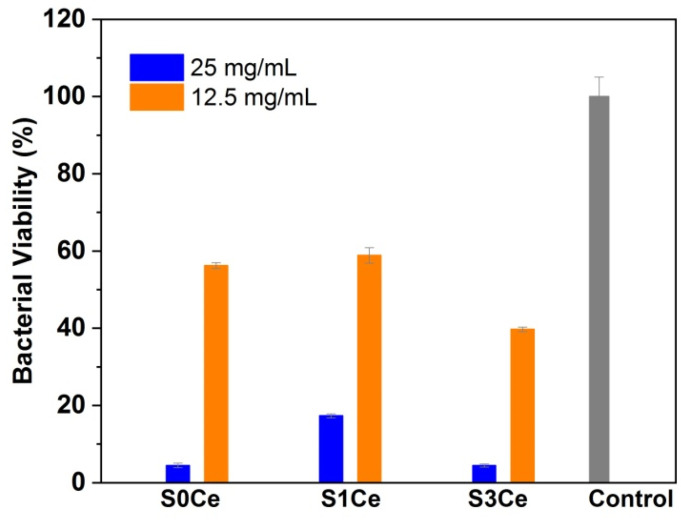
The effect of different concentrations of the samples on the *S. aureus* viability at 24 h. Results are shown as mean ± deviation standard, *n* = 3.

The antibacterial activity of the obtained scaffolds was investigated toward the Gram—positive reference strain *S. aureus* (ATCC 25923). Figure 8 shows the bacterial viability after 24 h of contact with 12.5 mg/mL and 25 mg/mL, respectively, of samples. All compositions showed antibacterial activity against *S. aureus.* A significant decrease in the bacterial viability at the highest concentrations of sample tested, of 25 mg/mL was observed. The highest antibacterial activity was obtained for the S3Ce sample.

The antibacterial activity of the cerium is controversial, and the mechanism underlying its antibacterial activity is still under study. The antibacterial activity of the cerium compounds is related to the inhibition of the oxidation and assimilation of glucose and endogenous respiration [47]. Recently, studies suggest that the antibacterial activity of cerium-containing glasses is influenced by glass composition, cerium amount, and morphology. A higher antibacterial activity was observed if the concentration of cerium oxide was in the 5−10 mol% range rather than 1 mol% [18]. Youness et al. [48] reported that the increase in cerium content in phosphate glasses enhanced their antibacterial activity against *E. coli* and *S. aureus*. Raimondi et al. [49] studied the antibacterial activity of cerium doped BGs up to a 5.3 mol% content and observed that this activity is not directly associated with the presence of cerium, and it is dependent on the change of pH due to glass dissolution. Goh et al. [50] reported the lack of antibacterial activity of polylactic acid/chitosan nanofibers decorated with cerium-containing bioactive glasses against *E. coli*, probably due to the slow release of ions from the glass composition and/or the small quantity of the glass adsorbed onto the nanofibers.

**Figure 9:**
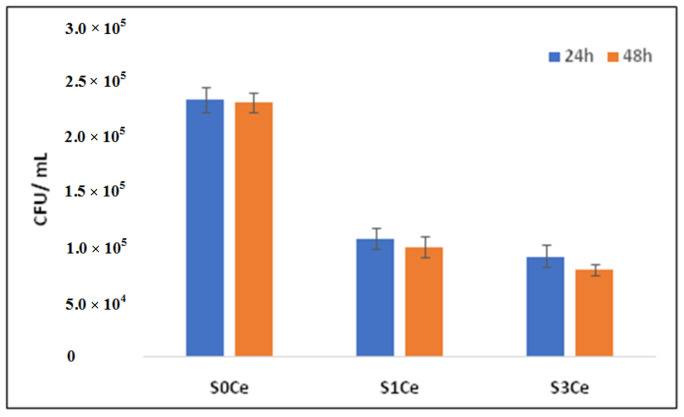
Bacterial viability (CFU/mL) after cultivation in the presence of samples loaded with VAN.

Moreover, the bacterial viability was evaluated after cultivation in the presence of scaffolds loaded with VAN. The results obtained (Figure 9) suggested an intrinsic antimicrobial effect of the samples similar to the data reported in the literature [49]. It was interesting to note a strong decrease in the microbial cells viability and ability to develop colonies in the case of bacteria seeded on S1Ce and S3Ce. Hence, our results indicate an enhancement of the antimicrobial activity exhibited by scaffolds loaded with vancomycin. Our data suggest a potential synergic effect due to the glass dissolution, cerium addition, and antibiotic release.

### 3.6. In Vitro Release Profiles and Kinetic Model

**Figure 10:**
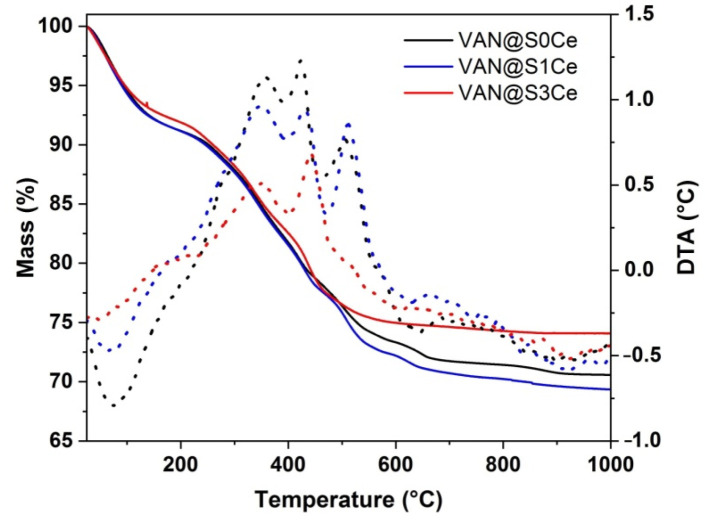
TGA-DTA analyses of VAN-loaded samples.

Thermogravimetric analyses (TGA) coupled with differential thermal analyses (DTA) were used to characterize the matrices and drug-loaded samples. All matrices exhibit very good thermal stability up to 1000 °C, with negligible mass loss (<1%, within the apparatus drift). In contrast, several mass loss events can be noticed for the VAN-containing samples (Figure 10). A 5–10% mass loss occurs on heating up to 180 °C for all drug-containing samples. This loss is accompanied by an endothermic effect, thus representing water loss (as the drug loading solvent). The combustion of vancomycin occurs through multiple exothermic events up to a temperature of around 900 °C. The VAN weight fraction was computed based on the total mass loss excluding the water contribution (Table 3). All samples exhibit a similar VAN fraction, of 20.3 ± 2.0 wt.%.

**Figure 11:**
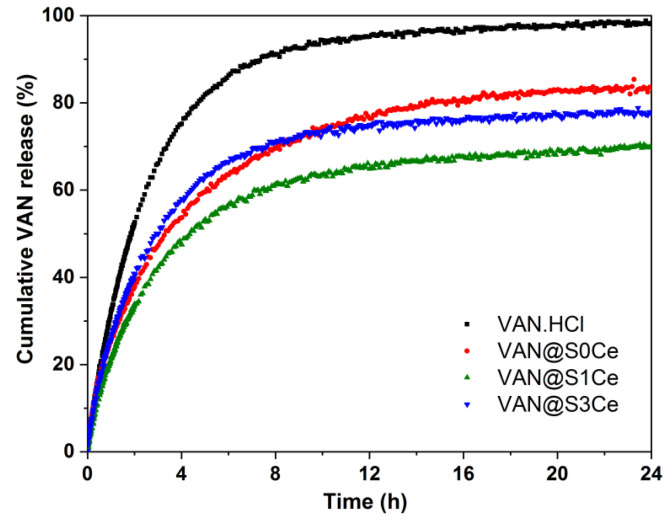
Cumulative drug release profiles of the samples in comparison with the dissolution of vancomycin hydrochloride.

The in vitro VAN release profiles were obtained at 37 °C, in 0.1 M phosphate buffer solution, pH 7.4, and they were compared to the vancomycin hydrochloride dissolution curve (Figure 11). All the samples show a two-step release profile, consisting of an initial fast, a burst stage, followed by a gradual, sustained release stage. The pure drug reaches 99% cumulative release after 24 h. The release kinetics form the inorganic carriers are slower in all cases. The higher cumulative release values are obtained for the carriers without Ce than the matrices containing Ce (Figure 10).

Each release profile was fitted with a first order kinetics, as well as a three-parameter model, first proposed by Zenget al. for nanocarriers [51]. In all cases, the three-parameter model provided a much better fit of the experimental data, even when accounting for the number of variables used (i.e., higher adjusted R^2^); therefore, it was the model used for explaining the VAN release kinetics. This model consists of three processes assumed to follow first order kinetics: adsorption and desorption processes of the drug molecules present on the carrier surface, which form an equilibrium and the transfer of the desorbed molecules into the release medium. The adsorption, desorption, and transfer processes are characterized by the *k_ads_*, *k_des_*, and *k_tr_* kinetics constants, respectively, while a Gibbs free energy parameter, Δ*G*, can be associated to the adsorption/desorption equilibrium. Since the transfer and diffusion process is much faster than the surface adsorption/desorption equilibrium, the release rate during the initial burst stage is proportional to *k_tr_*, as the fraction of drug molecules desorbed at *t* = 0 is released during this stage. The desorption rate constant becomes rate limiting during the sustained release stage. The Δ*G* parameter indicated the ratio of the amount drug released during the burst to the sustained release stages [52]. The analytical expression of the three parameters model is presented in Equation (2).

(2) $$\frac{m\left(t\right)}{m\left(\infty \right)}=\frac{\lambda_{2}\left(k_{tr}-\lambda_{2}\right)}{\left(k_{ads}+k_{des}\right)\left(\lambda_{1}-\lambda_{2}\right)}\left(1-e^{-\lambda_{1}t}\right)+\frac{\lambda_{1}\left(\lambda_{1}-k_{tr}\right)}{\left(k_{ads}+k_{des}\right)\left(\lambda_{1}-\lambda_{2}\right)}\left(1-e^{-\lambda_{2}t}\right)$$

where: $m\left(t\right)$ and $m\left(\infty \right)$ are the cumulative amount of drug released up to time *t* and the total drug amount, respectively, and

$$\lambda_{1,2}=\left[\left(k_{tr}+k_{ads}+k_{des}\right)\pm \sqrt{{\left(k_{tr}+k_{ads}+k_{des}\right)}^{2}-4k_{tr}k_{ads}}\right]/2.$$

Adjusted R^2^ values higher than 0.994 were obtained in all cases (Table 3), indicating that this model fits well with the experimental release data. The dissolution of the drug was also fitted with the three parameters model, for comparison reasons only. The transfer rate constant, *k_tr_*, is similar for all samples. In contrast, the desorption rate, *k_des_*, which is proportional to the sustained release rate, is reduced by 3.3–6.7times for the Ce-containing carriers in comparison with the samples without Ce (Table 3). Ce doping therefore significantly prolongs the duration of the sustained release stage by decreasing the VAN desorption kinetics rate during this stage. The Δ*G* parameter follows the same trend, with lower values for the Ce-containing samples, indicating that a smaller amount of drug is released during the burst stage for S1Ce and S3Ce, in comparison with the S0Ce sample.

## 4. Conclusions

Cerium-containing MBGs-based scaffolds were obtained using PMMA as a sacrificial template.

UV-Vis analysis highlighted the presence of cerium in both oxidation states Ce^4+^/Ce^3+^, with the Ce^4+^/Ce^3+^ ratio of ~1/3.25, for S1Ce and of ~1/1.51, for S3Ce.

The nitrogen adsorption–desorption isotherms confirmed the presence of mesopores with an average value of ~4 nm for S0Ce and S3Ce, whereas for S1Ce the average value was ~4.6 nm. Lower values of the BET surface area were obtained without affecting bioactivity and drug delivery efficiency. Besides mesoporosity, macroporosity was also underlined by SEM analysis.

The formation of the HA layer over the surface of the investigated samples was confirmed by the results of XRPD, FTIR, and SEM analyses.

All the prepared scaffolds exhibit antibacterial activity, including that without cerium and regardless of the amount of cerium. An enhancement of the antibacterial activity was observed in the case of scaffolds loaded with vancomycin.

In vitro cytocompatibility evaluation confirmed no cell cytotoxic activity on L929 cells.

The investigated materials can also serve as carriers for the antibiotic vancomycin hydrochloride, with drug loading fractions of 20 wt.%. All experimental release profiles consist of a two-stage process: a fast, burst release stage, followed by a sustained release phase. This type of release profile can provide both an initial strong antibiotic dose during the first 6h and a continued multi-day sustained release. Cerium-doping results in a decrease in the vancomycin-sustained release rates, thus prolonging the duration of this phase. The sustained release kinetics rate is reduced by 3.3–6.7 times in comparison with the un-doped scaffolds, depending on cerium content.

From the obtained results, it can be concluded that the prepared scaffolds are potential candidates for bone regeneration applications.

## Figures and Tables

**Table 1:** The position of the maximum of the absorption band of PMMA, Ce^4+^, and Ce^3+^ of the Gaussian bands, in the S1Ce and S3Ce samples, with their corresponding percentages.

Sample	PMMA-MBGs	λ_abs,Ce_^4+^	λ_abs,Ce_^3+^	Impurity/Others
S1Ce	225 nm (4.32%)	256 nm (19.79%)	322 nm (64.39%)	500–750 nm (11.50%)
S3Ce	228 nm (5.48%)	267 nm (34.85%)	341 nm (52.72%)	500–750 nm (6.95%)

**Table 2:** Textural parameters of the S0Ce-S3Ce set samples.

Sample	S_BET_ (m^2^/g)	Average Pore Diameter (nm)	Total Pore Volume (cm^3^/g)
S0Ce	96.98 ± 0.43	4.07	0.10
S1Ce	136.96 ± 0.29	4.67	0.15
S3Ce	113.36 ± 0.78	3.93	0.10

**Table 3:** VAN weight fraction, kinetic fitting parameters, and adjusted *R*^2^ values.

Sample	%VAN (wt.%)	Δ*G* (10^−21^ J/Molecule)	*k_tr_* (h^−1^)	*k_des_* (h^−1^)	*k_ads_* (h^−1^)	*Adj. R*^2^ (−)
VAN.HCl	100	10.61	0.421	0.087	0.007	0.9998
VAN@S0Ce	20.8	2.67	0.462	0.050	0.027	0.9957
VAN@S1Ce	22.0	1.99	0.404	0.015	0.009	0.9985
VAN@S3Ce	18.0	3.89	0.435	0.016	0.006	0.9981

## Data Availability

The data presented in this study are contained within the article.
